# Full Validation and Application to Clinical Research of a High-Performance Liquid Chromatography Method for the Assessment of Urinary 3-Indoxyl Sulfate in Pediatric Patients with Hematopoietic Stem Cell Transplant

**DOI:** 10.3390/mps7040064

**Published:** 2024-08-19

**Authors:** Christian Ezequiel Olivetti, María Florencia Fernández, Jana Stojanova, Silvina Ruvinsky, Andrea Mangano, Paula Schaiquevich

**Affiliations:** 1 Unit of Innovative Treatments, Hospital de Pediatria JP Garrahan, Buenos Aires CP1245, Argentina; christianolivetti@yahoo.com.ar; 2 Unit of Molecular Virology and Epidemiology, Hospital de Pediatria JP Garrahan, Buenos Aires CP1245, Argentina; mariaflor_10@hotmail.com (M.F.F.); amangano@garrahan.gov.ar (A.M.); 3 Department of Clinical Pharmacology, Toxicology, St. Vincent’s Hospital Sydney, Sydney 2007, Australia; 4 Research Department, Hospital de Pediatria JP Garrahan, Buenos Aires CP1245, Argentina; sruvinsky@garrahan.gov.ar; 5 National Scientific and Technical Research Council, CONICET, Buenos Aires CP1414, Argentina

**Keywords:** high performance chromatography, 3-indoxyl sulfate, pediatrics, urine, dysbiosis

## Abstract

3-indoxyl sulfate (3-IS) results from a hepatic transformation of indole, a tryptophan degradation product produced by commensal gut bacteria. The metabolite has shown promise as a biomarker of dysbiosis and clinical outcomes following hematopoietic stem cell transplant (HSCT) in adults. Nonetheless, there is a paucity of data regarding microbiome health and outcomes in the pediatric HSCT setting. We developed and thoroughly validated an affordable high-performance liquid chromatography/fluorescence detector (HPLC-FLD) method to quantify 3-IS in urine for use in the pediatric setting. Chromatographic separation was achieved on a C18 column (250 × 4.6 mm × 5 μm) with a mobile phase consisting of pH 4.0 acetic acid-triethylamine buffer and acetonitrile (88:12, *v*/*v*), eluted isocratically at 1 mL/min. 3-IS fluorescence detection was set at excitation/emission of 280 and 375, respectively. The method was fully validated according to FDA-specified limits including selectivity, linearity (0.10 to 10.00 mg/L, r^2^ > 0.997), intra- and inter-day accuracy, and precision. 3-IS stability was confirmed after three freeze–thaw cycles, for short- and medium-term on a benchtop and at 4 °C and for long-term up to 60 days at −20 °C. The validated method was used to quantify 3-IS in urine samples from HSCT pediatric patients.

## 1. Introduction

The human gut harbors a large collection of bacteria, fungi, and viruses known as the gut microbiota. The bacterial phyla *Firmicutes*, *Bacteroidetes*, *Actinobacteria*, and *Proteobacteria* predominate. When all genes and gene products of gut microbes are considered, we refer to the microbiome, and emerging evidence supports its critical role in human health and disease [1,2]. Disruption of gut microbiome homeostasis, referred to as dysbiosis, can influence several disease processes including deregulation of the immune system, inflammation, and infection contributing to gastrointestinal alterations [3,4,5].

The evidence to date predominantly reflects work in adult patients; thus, methods to detect dysbiosis in pediatric HSCT patients are important for both research and clinical applications.

Genomic characterization of the microbiome is complex, time-consuming, and costly. If clinical utility can be demonstrated for a given biomarker of dysbiosis, an affordable and less technically complex assay method would enable swift clinical translation.

Hematopoietic stem cell transplantation (HSCT) is an essential therapy for many hematologic malignancies and immune deficiency syndromes; however, some patients experience gastrointestinal graft-versus-host disease (GI GVHD), which can be life-threatening [6,7]. Several preclinical and clinical studies have shown GI GVHD is associated with dysbiosis, which subsequently contributes to intestinal inflammation [8,9]. Intensive chemotherapy, body irradiation, and prophylactic antibiotic treatment provided in peri-conditioning regimens can also affect the intestinal epithelia and microbiota [10,11,12,13]. Moreover, HSCT conditioning regimens, as well as associated therapies and procedures, can impact the composition of the microbiome. In adult HSCT recipients, reduced microbiome composition and diversity have been associated with a higher risk of GVHD, as well as relapse or mortality rates primarily due to GI GVHD [9,14,15,16]. Recent studies have provided preliminary evidence for a relationship between dysbiosis and outcomes in pediatric HSCT patients, although these studies have typically involved small patient cohorts [17,18,19]. It is thus crucial to characterize temporal changes in microbiome composition, as timely detection of dysbiosis may help identify individuals at risk and provide the most convenient intervention.

Genomic characterization of the microbiome is complex, time-consuming, and costly. If clinical utility can be demonstrated for a given biomarker of dysbiosis, an affordable and less technically complex assay method would enable swift clinical translation.

Tryptophan-derived indole compounds produced by commensal intestinal bacteria play a role in the communication between the microbiota and host, influencing various physiological processes, including intestinal epithelial integrity, protection from inflammation, immunologic homeostasis, and intestinal tolerance [20,21]. The predominant indole metabolite in humans, 3-indoxyl sulfate (3-IS), results from the hepatic transformation of indole in the host and is thereafter renally excreted [22]. Low urine concentrations of 3-IS appear to reflect dysbiosis and have been identified as a significant risk factor for transplant-related mortality attributed to GI GVHD in adult HSCT recipients [23,24,25]. Nonetheless, urinary 3-IS as a biomarker of dysbiosis and HSCT outcome has not been evaluated in children to date.

Bioanalytical methods to quantify 3-IS in urine typically involve instruments with mass spectrometry detection, which are not always available in middle-income countries due to high operational costs [23,26,27,28,29]. While HPLC-UV-based methods have been reported, they represent low selectivity and sensitivity [30,31,32]. The physicochemical properties of 3-IS, which include an XlogP_3_ of 1.3 and fluorescence characteristics related to structural similarities with tryptophan, make it an excellent candidate for fluorescence detection (FLD) [33]. RP-HPLC-FLD methods published to date lack thorough validation at international standards on bioanalytical methods, especially for quantification of urinary 3-IS in the pediatric context [34,35,36].

In this work, we developed and validated a sensitive, robust, and affordable HPLC-FLD method to quantify 3-IS in urine. We applied the method to urine samples from pediatric HSCT recipients and demonstrated its suitability for this application. If 3-IS is shown to have clinical utility, which is being evaluated in ongoing clinical research currently performed at our hospital, this method could be suitable for clinical use.

## 2. Materials and Methods

### 2.1. Chemicals and Reagents

Reference standards for 3-indoxyl sulfate potassium salt (1H-indol-3-yl hydrogen sulfate monopotassium salt) and metoprolol tartrate (internal standard) were purchased from Merck (Darmstadt, Germany) and Sigma-Aldrich (Massachusetts, MA, USA), respectively. Ammonium phosphate was purchased from Merck (Darmstadt, Germany). HPLC grade acetonitrile (AcN) and methanol (MeOH), 99.7% glacial acetic acid, and Triethylamine (TEA) were purchased from Sintorgan (Buenos Aires, Argentina) and sodium hydroxide from Biopack (Buenos Aires, Argentina), phosphate monohydrate (NaH_2_PO_4_·H_2_O) from Merck (Darmstadt, Germany), and disodium dibasic phosphate (Na_2_HPO_4_) from Anedra (Argentina), respectively. Water was purified using a Milli-Q-system (Millipore Corporation, Billerica, MA, USA).

### 2.2. Instrumentation and Chromatographic Conditions

An HPLC alliance e2695 system (Waters Corporation, Mildford, CT, USA) consisted of a quaternary pump, an online degasser, and an autosampler coupled with a Waters 2475 fluorescence detector set at λ_excitation_ of 280 nm. Both 3-IS and the metroprolol emission wavelength were set at 375 nm, but, as the fluorescence intensity of metoprolol is higher at 300 nm, we also quantified the internal standard using this second emission wavelength. Program setting, data acquisition, and processing were performed with Empower 3 (Waters Corporation, Mildford, CT, USA) software.

Chromatographic separation was achieved with isocratic elution using a Symmetry ODS C18 column (250 mm length and 4.6 mm id, particle size 5 μm, 100 Å) equipped with a C18 Security Guard pre-column (Phenomenex, Torrance, CA, USA) and maintained at 25 °C.

The mobile phase consisted of 50 mM glacial acetic acid adjusted to pH 4.0 with 5 M sodium hydroxide–43.4 mM TEA buffer, which was premixed in a reservoir bottle and then mixed with ACN in a proportion of 88:12 (*v*/*v*). The mobile phase was filtered through a 0.22 μm filter and degassed with an ultrasonic bath and delivered at a flow rate of 1 mL/min. Samples were kept at 4 °C in the autosampler, and the injection volume was 20 μL. After each injection, the system was programmed to be washed with a mixture of MeOH/water (50:50). The run time was 40 min. To assess the best mobile phase composition, peak asymmetry was calculated using the following formula:

$$A_{s}=\frac{W_{0.05}}{2d}$$

in which *A_s_* is the symmetry factor, *W*_0.05_ the width at the 5% peak height, and *d* the distance between the perpendicular dropped from the peak maximum and the leading edge of the peak at one-twentieth of the peak height. To satisfy the acceptance criteria, the symmetry factor should be less than 1.5.

### 2.3. Stock and Working Solutions

Stock solutions of 1 mg/mL of both 3-IS and metoprolol were prepared in HPLC grade MeOH. As 3-IS is an endogenous metabolite, urine from healthy volunteers cannot be used for the blank matrix. Thus, artificial urine was prepared by dissolving 31.25 mmol of sodium monobasic phosphate monohydrate (NaH_2_PO_4_.H_2_O) and 6.25 mmol of disodium dibasic phosphate (Na_2_HPO_4_) in 250 mL of purified water, producing a 150 mM phosphate buffer that was adjusted to pH 5.8 [37]. The final solution was filtered through a 0.45 μm polypropylene filter. Also, a solution of artificial urine supplemented with ammonium was prepared as described for artificial urine and supplemented with ammonium at a final concentration of 19 mmol/L [38].

Calibrators and quality controls (QC) were prepared by spiking 3-IS and metoprolol into artificial urine. A six-point calibration curve consisted of the following concentrations: 0.10, 0.25, 0.50, 1.00, 5.00, and 10.00 mg/L of 3-IS; metoprolol was spiked to result in 10 mg/L in all cases. The concentration range was defined based on 3-IS urine concentrations reported in adult patients with HSCT and considering a 1:20 dilution [23,24,39]. QC samples were separately prepared to represent low, middle, and high levels: L-LOQ of 0.10 mg/L, M-QC of 1.00 mg/L, and U-LOQ of 10.00 mg/L of 3-IS.

### 2.4. Human Sample Preparation

For urine analysis, a 1 mL aliquot urine sample from healthy volunteers and pediatric patients with HSTC was transferred to a 1.5 mL polypropylene tube and centrifuged at 16,000× *g* for five minutes. The supernatant was diluted 1:20, incorporating a 50 μL aliquot of the sample, 10 μL of a 1 mg/L metoprolol methanolic solution, and 940 μL of phosphate buffer. Then, the diluted sample was centrifuged and filtered through a 0.22 μm Nylon filter.

### 2.5. Assay Validation

The Food and Drug Administration (FDA), and the European Medicines Agency (EMA) guidelines were followed to validate the method, specifically methodological guidance for analytes that are endogenous in the matrix being evaluated [40,41].

#### 2.5.1. Selectivity and Sensitivity

To evaluate selectivity, artificial urine was spiked and analyzed at the L-LOQ level, checking the absence of interference at the retention time of the 3-IS. To corroborate the selectivity of the method in the biological matrix, we compared the absorption/emission spectra of the M-QC at the retention time of 3-IS for two urine samples from healthy volunteers. Signal interference of <20% of the L-LOQ was considered acceptable.

As 3-IS is an endogenous compound, it is difficult to find a suitable blank matrix for evaluations. We, therefore, performed evaluations involving artificial urine with ammonium and urine from a pediatric HSCT patient expected to have low 3-IS concentrations (receiving antibiotics due to neutropenia fever).

In the case of the effect of ammonium, this compound is associated with autofluorescence of human urine at a short UV (250–300 nm) excitation wavelength. Moreover, others have shown that once human urine is diluted, ammonium does not impact the fluorescence intensity of 3-IS [38]. To corroborate this in our experimental design involving artificial urine, we evaluated the 3-IS signal at two QC concentrations in artificial urine with and without 1 mM ammonium, representing a 1/20 dilution and considering a physiological ammonium concentration of 19 mmol/L [38].

For the second evaluation, we used the urine from a pediatric HSCT patient who received antibiotics and thus was expected to have minimal 3-IS (below the L-LOQ) in his urine, and after 1/20 dilution, we spiked with 3-IS and internal standard to produce a calibration curve for comparison with the one produced using artificial urine.

For sensitivity, the L-LOQ was defined as the concentration that could result in a signal-to-noise (S/N) ratio of ≥5 in artificial urine solution.

#### 2.5.2. Linearity

The linearity of the calibration curves was tested by analyzing six quality control solutions (QC) in triplicates on four independent runs. The peak area (AUC) of the triplicate measurements was plotted against the nominal concentration and fitted using the least squares methods with a 1/x^2^ weighting. Back calculated concentrations were required to be within ±15% of nominal concentrations and ±20% for the L-LOQ. At least 75% of the QC levels were required to fulfill these criteria in each run. The correlation coefficient of each standard curve obtained with linear regression analysis was required to be ≥0.98 in all cases.

#### 2.5.3. Intra- and Inter-Day Precision and Accuracy

The intra- and inter-day accuracy and precision of the method were evaluated in five replicates of the L-LOQ (0.1 mg/L), M-QC (1.00 mg/L), and U-LOQ (10.00 mg/L) measured on three consecutive days. QC samples were interpolated in a fresh calibration curve and the calculated concentration was compared to the nominal value. Accuracy was calculated as the percent ratio of the measured concentration to the nominal value, while precision (coefficient of variation, CV%) was determined as the ratio of the standard deviation to the mean concentration.

Also, intra- and inter-day accuracy and precision of the method were performed in urine samples from two healthy volunteers. These samples were spiked with 3-IS QCs at three levels (corresponding at 0.10, 1.00, and 10.00 mg/L) and metoprolol 10 mg/L as IS; they were quantified in quintuplicates on 3 consecutive days, and the accuracy and precision were calculated as described before. Acceptance criteria for accuracy were expected between 85% and 115% and for precision <15% except for the L-LOQ < 20%.

#### 2.5.4. Recovery

Per FDA guidelines for endogenous compounds, recovery was evaluated in two urine samples from healthy volunteers that were spiked with 100 µL of 3-IS QC (1, 5, and 10 mg/L) to 900 µL of urine (final concentrations in diluted urine: 0.1, 0.5, and 1 mg/L). Analyses were performed in triplicate, and the mean was considered. The 3-IS concentration was determined with interpolation in a calibration curve, and recovery was calculated as follows:

$$Recovery\;\%=\frac{\left({mean\;3IS\;Conc}_{spiked\;}-{mean\;3IS\;Conc}_{\;non-spiked}\right)}{\left(mean\;spiking\;concentration\right)}\times 100$$

#### 2.5.5. Stability

Stability was tested after three −20 °C freeze–thaw cycles, running five QC replicates. Short- and medium-term stability was evaluated at room temperature and light (benchtop condition) and 4 °C for 1, 2, 4, and 6 h and 1 and 2 weeks. Long-term stability was assessed following 1- and 2-month storage at –20 °C.

### 2.6. Clinical Research Samples

The validated bioanalytical assay was used to quantify 3-IS in urine samples from pediatric HSCT recipients after informed consent was obtained from parents or legal guardians. This study was approved by the Institutional Review Board at Hospital de Pediatria JP Garrahan (protocol #1452) and was conducted in accordance with the International Conference on Harmonization Good Clinical Practice guidelines and the Declaration of Helsinki. Samples were collected in polypropylene sterile containers and a fraction was separated for urinary creatinine measurement using the compensated Jaffe colorimetric method (Cobas 6000, Roche). Samples were stored at −80 °C until analysis. Concentrations of 3-IS were normalized to urinary creatinine levels according to the following formulae [42,43]:

$$\frac{{µmol}_{3-IS}}{{mmol}_{creatinine}}=\frac{100\frac{\mathrm{mL}}{\mathrm{dL}}}{\left(\frac{\left[{Urinary\;creatinine}_{\mathrm{mg}/\mathrm{dL}}\right]}{{Creatinine\;MW}_{\mathrm{mg}/\mathrm{mmol}}}\right)}*\left(\frac{\left\lfloor {3IS\;urinary\;concentration}_{µg/\mathrm{mL}}\right\rfloor }{{3IS\;MW}_{µg/µmol}}\right)$$

## 3. Results

### 3.1. Assay Development and Validation

#### 3.1.1. Chromatographic Conditions

Optimal chromatographic conditions were achieved using a mobile phase comprising 50 mM acetic acid adjusted to pH 4.0 with TEA buffer and AcN (88:12, %*v*/*v*). TEA was critical for an acceptable 3-IS peak shape, especially for urine samples from healthy volunteers, likely due to reduced peak tailing caused by the uncoated silanol groups of the stationary phase. The addition of TEA resulted in a symmetry factor of 1.235 compared to 1.575 obtained using the mobile phase without TEA. 3-IS fluorescence intensity in phosphate buffer (artificial urine) was similar to that in phosphate buffer supplemented with 1 mM ammonium (Table 1).

**Figure 1:**
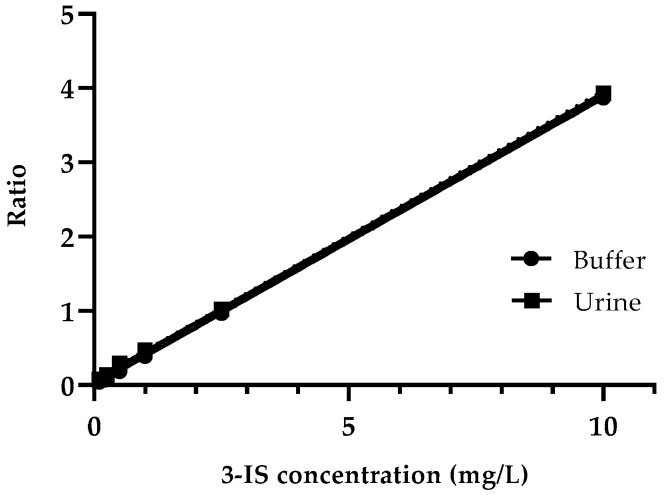
Calibration curve in artificial urine and diluted urine from a neutropenic patient. Calibration curve obtained in urine: slope = 0.3866 (SE: 0.0016; 95%CI: 0.383–0.390); calibration curve obtained in artificial urine: slope = 0.386 (SE: 0.0029; 95%CI: 0.386–0.387).

The calibration curves produced using diluted urine from a patient with a low 3-IS concentration and artificial urine were superimposable and similar (Figure 1; Slope test, *p* = 0.98).

#### 3.1.2. Selectivity and Sensitivity

**Figure 2:**
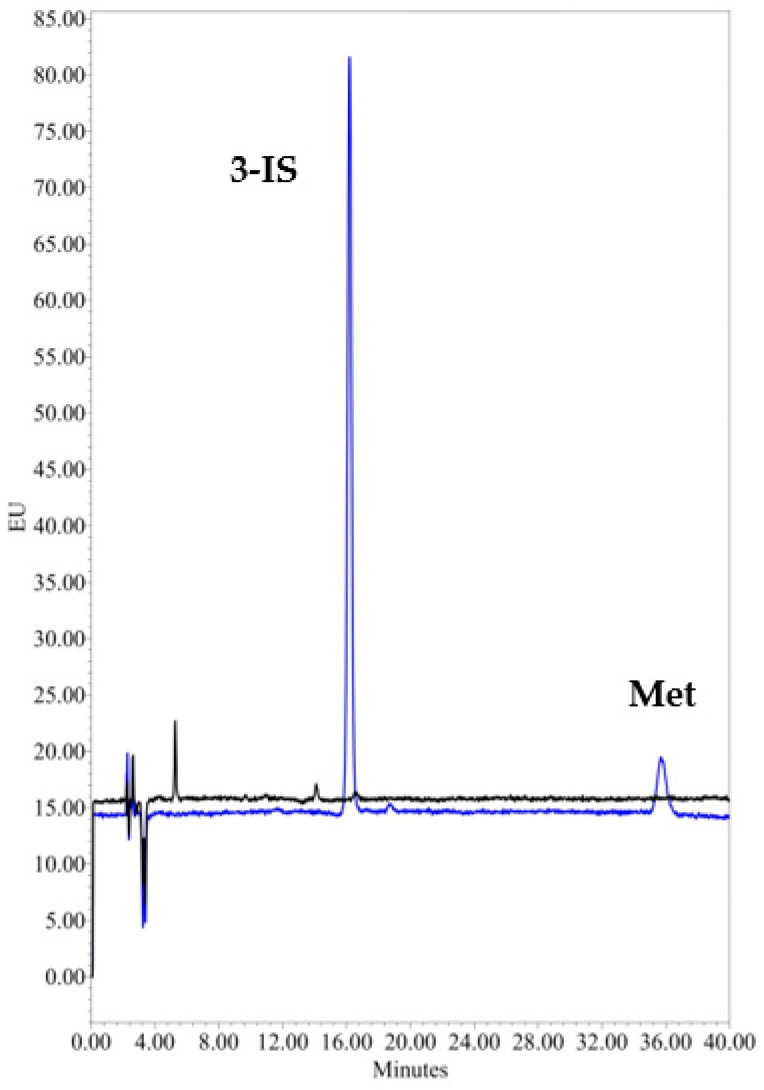
Overlay of artificial urine and a representative chromatogram of 3-IS at the L-LOQ and the internal standard, metoprolol. Black line: artificial urine; Blue line: L-LOQ.

Analysis of five replicates of the L-LOQ sample in artificial urine showed excellent 3-IS sensitivity; precision and accuracy at a concentration of 0.1 mg/L were 0.47% and 105.0%, respectively, with a signal-to-noise (S/N) ratio of >50 (Figure 2, LLO-Q vs. artificial urine).

**Figure 3:**
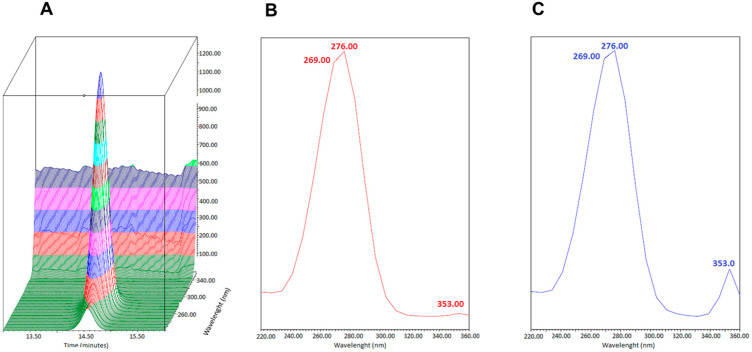
Absorption spectra at the retention time of 3-IS of (**A**) 3-IS U-LOQ (3D spectra), (**B**) 3-IS U-LOQ (2D spectra), and (**C**) 2D absorption spectra of a urine sample from a healthy volunteer.

**Figure 4:**
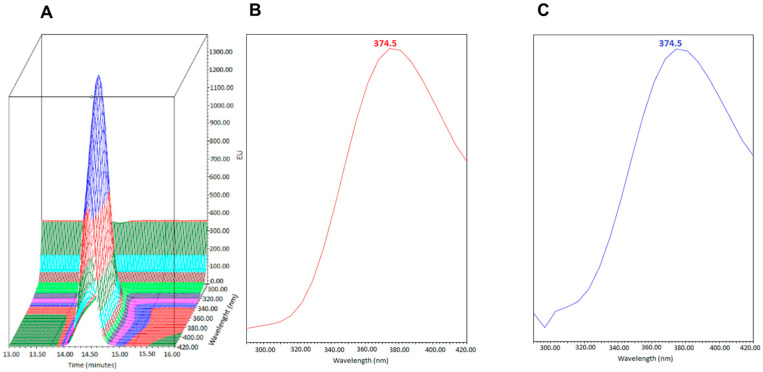
Emission spectra at the retention time of 3-IS of (**A**) 3-IS U-LOQ (3D spectra), (**B**) 3-IS U-LOQ (2D spectra), and (**C**) 2D emission spectra of a urine sample from a healthy volunteer.

The selectivity of the chromatographic method was assessed by comparing the absorption/emission spectra at the 3-IS retention time by comparing urine samples from healthy volunteers to the U-LOQ solution prepared in artificial urine. Figure 3 and Figure 4 show that the absorption/emission of the two were superimposable, indicating that no other analytes co-eluted at 3-IS retention time.

#### 3.1.3. Linearity

The intercepts, slopes, and correlation coefficients of four different calibration curves that each consisted of six calibrators with concentrations ranging from 0.10 to 10.00 mg/L are shown in Table 2. According to the relation between the absolute % residual error and total residues, a weighted 1/x^2^ linear regression was the best fit for the calibration curve and resulted in a 3.84% total error. Moreover, the back-calculated biases of the four calibration runs were from −18.6% for L-LOQ values up to 9.8% for U-LOQ, whereas the correlation coefficient (r^2^) ranged between 0.997 and 0.999. Altogether, the assay was considered to have a linear response in the full assayed range.

#### 3.1.4. Accuracy and Precision

Accuracy and precision were calculated for each QC level on each day and between days in artificial urine (Table 3). Intra-day accuracy ranged from 100.1% to 112.6%, and within-run precision ranged from 0.2% to 1.5%. Inter-day accuracy ranged 100.5–108.4%, and precision was between 1.7% and 3.7%. Overall, accuracy and precision satisfied FDA and EMA criteria [40,41].

#### 3.1.5. Recovery

Recovery was calculated using urine from two healthy volunteers following spiking with three QC solutions of 3-IS according to the FDA guidelines criteria [40,41]. The recovery evaluations using urine from healthy volunteers were acceptable (Table 4).

Creatinine normalized values were 28.66 µmol _3-IS_/mmol _creatinine_ and 78.75 µmol _3-IS_/mmol _creatinine_ for volunteers 1 and 2, respectively, based on urinary creatinine values of 1145.3 and 357.7 mg/L, respectively.

#### 3.1.6. Stability

After three freeze–thaw cycles, percentage values for L-LOQ, M-QC, and U-LOQ relative to the first day were 109.6%, 102.9%, and 97.5%, with a precision of 1.14%, 0.46%, and 0.17%, respectively. Moreover, 3-IS remained stable after three freeze–thaw cycles in urine from human volunteers, with accuracy between 96.9 and 109.6% and precision between 0.5 and 1.2%.

Short- and medium-term 3-IS stability results at various conditions are presented in Table 5. As shown in this table, 3-IS was stable for up to 14 days under both benchtop and refrigerated conditions.

All results fall within 85–115% of the nominal value following short-, medium- and long-term storage at various conditions, fulfilling the FDA guideline criteria (Table 5 and Table 6).

### 3.2. Clinical Application

We tested the applicability of our analytical method in a pediatric setting using urine samples from five patients who received HSCT. Demographic, clinical, and pharmacological data are depicted in Table 7. Normal and expected values for 3-IS urine concentrations are currently unknown in the pediatric HSCT context.

**Figure 5:**
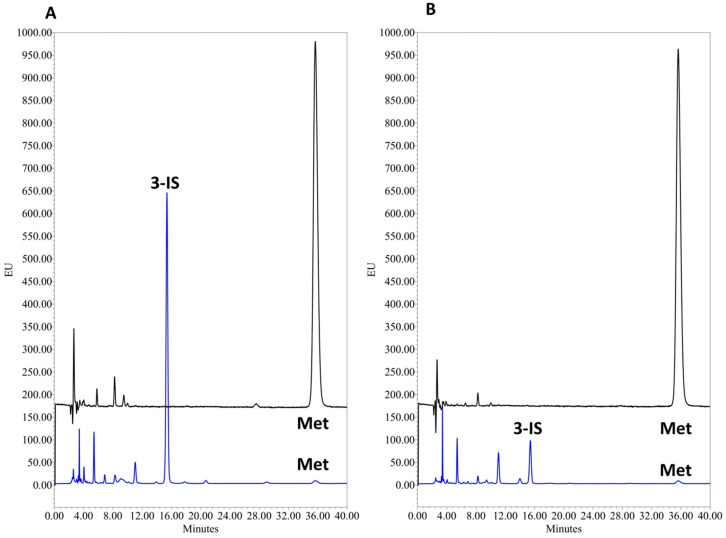
Overlay of chromatograms of urine samples spiked with internal standard from pediatric patients at 375 and 300 nm emission wavelengths. Chromatograms of patient 1 (**A**) and patient 2 (**B**). Blue lines: chromatograms of urine samples spiked with metoprolol 10 mg/L from two representative patients at 375 nm emission wavelength. Black line: Chromatograms of urine samples from two representative patients at 300 nm emission wavelength. Abbreviations: 3-IS3-indoxyl sulfate; Met: metoprolol.

The chromatograms of two representative patients are shown in Figure 5. The median 3-IS urine concentration obtained from the samples from the five patients was 8.10 mg/L (range: 2.0–31.9 mg/L). In terms of creatinine normalized levels, 3-IS urine concentrations ranged from 3.4 to 67.9 μmol_3-IS_/μmol_creatinine_.

## 4. Discussion

We developed a novel and simple HPLC-FLD method to quantify 3-IS, and we have shown that it is suitable for use in the pediatric HSCT setting. While simple, the method is robust and has been fully validated to meet the standards of international bioanalytical guidelines. This method is more affordable compared to those employing mass spectrometry, making it especially suitable for resource-limited settings.

Loss of intestinal microbiota diversity correlates with poor HSTC outcomes, including the risk and outcomes of serious complications such as GI GVHD [14,17,19]. The evidence to date predominantly reflects work in adult patients; thus, methods to detect dysbiosis in pediatric HSCT patients are important for both research and clinical applications.

Tryptophan degradation by commensal gut bacteria produces indole, which is reabsorbed and undergoes hepatic transformation to 3-IS, which is, in turn, excreted in urine [22]. A low urinary 3-IS concentration has been proposed as a promising biomarker for dysbiosis, and several studies have demonstrated associations with clinical outcomes [23,44,45]. These publications reflect studies in adult patients; urinary composition in pediatric HSCT patients may differ from adults and could influence requirements for a successful assay method. Furthermore, the suitability of 3-IS as a biomarker of dysbiosis in the pediatric setting remains to be evaluated.

Diverse methods for 3-IS quantification have been published, including LC-MS/MS, which requires expensive instruments that are hardly available in middle-income countries [22,26]. Others have developed HPLC methods that have typically involved diverse human and animal fluids but not urine [36,46,47,48]. Although HPLC-FLD methods have been published, validation at international bioanalytical standards was not performed. [34,36,38,47]. Importantly, urine from pediatric patients was not tested in these HPLC-FLD methods, an important point, as the sensitivity and specificality of the assay may differ in this context.

Modifications to the composition of the mobile phase previously reported by others were assessed [49]. Optimal retention time and peak separation were achieved using AcN 12% *v*/*v* and adding to the acetic acid buffer 43.4 mM TEA, which, although a small volume, critically improved the 3-IS peak shape. We assessed the possible modification of fluorescence intensity of 3-IS with ammonium, corroborating the finding that at high sample dilutions, this is not an issue [48].

The assay had a linear response in the range of 0.10 to 10.00 mg/L in processed and diluted samples. This concentration range was selected based on expected concentrations in urine samples from adult HSCT patients [23,25]. Importantly, intra- and inter-day accuracy and precision of QC samples, as well as urine specimens from healthy volunteers, satisfied the FDA and EMA guidelines’ criteria [40,41].

Collection of urine samples in the pediatric setting can be challenging even with strict protocols and standard operating procedures. For outpatients, samples may need to be transported. Thus, stability assessment of 3-IS in urine samples is vital to inform the reliability of results following diverse storage conditions. To this end, we developed stability studies intended to evaluate handling and storage conditions that are typical in the course of clinical care.

First, the stability of 3-IS in artificial urine and in healthy volunteers’ urine samples was tested after three freeze–thaw cycles of −20 °C, as these freezers are more common in hospitals *versus* −80 °C ultra-freezers. The stability of 3-IS at room temperature at different time intervals was also evaluated, as this is the usual condition for sample handling in most hospital bioanalytical laboratories. We also evaluated the short-term stability for up to 6 h and 14 days at 4 °C in case 3-IS instability was determined with the benchtop condition. 3-IS remained stable after the three freeze–thaw cycles in both artificial urine and urine samples from human volunteers and up to 14 days at both room temperature and 4 °C. Nevertheless, we recommend samples be stored at 4 °C whenever possible to prevent microbial growth in cases of urinary infections for staff safety. Importantly, samples can be stored at −20 °C for at least 2 months before 3-IS quantification. Altogether, we show that under all conditions evaluated, there was no significant degradation of 3-IS either in artificial urine or in healthy volunteer samples.

Finally, we assessed the performance of the assay in urine from five pediatric HSCT patients. We developed a method that requires simple sample preparation, as it only consists of centrifugation, dilution, and filtration before injection into the chromatographic system. This is of particular importance in the setting of sample processing at hospital laboratories. We observed a 15-fold difference between the highest and lowest 3-IS urine concentrations, which may be due to differences in conditioning regimens and concomitant antibiotics and the resulting impact on intestinal microbiota [15,25]. More detailed analyses of the impact of therapeutic agents, before and after therapeutic protocols are applied, are planned for future studies.

Limitations of the present method should be acknowledged. As 3-IS is an endogenous compound, there is a lack of a suitable blank matrix for use in selectivity and specificity assessments. We used artificial urine as a synthetic substitute and we acknowledge that it may not entirely reflect a variety of possible matrix effects that could affect analyte behavior in the method. We nonetheless verified that this matrix is a suitable surrogate by evaluating the effect of diluted ammonium and comparing the slopes of calibration curves prepared with artificial urine to those using urine with low 3-IS concentrations. In both cases, modifications in the fluorescence intensity of 3-IS were ruled out, supporting the use of artificial urine under the conditions we describe. The selectivity of the method was nevertheless demonstrated in analyses involving urine samples from healthy adult volunteers and pediatric patients.

## 5. Conclusions

A simple, robust, and affordable RP-HPLC-FLD method for the quantification of 3-IS in urine samples from children was developed and fully validated in accordance with international guidelines for bioanalytical method validation. Sample preparation is simple and does not require any special equipment, making the technique affordable for limited-resource settings. The accuracy and precision of the method were concordant with international regulatory guidelines, and the calibration range covered the expected concentration range for 3-IS in urine from pediatric HSCT patients. Finally, the assay performance was successfully tested to measure 3-IS urine concentrations from children undergoing HSCT. Overall, owing to its simplicity in sample preparation and analysis, this method is suitable for clinical application, even in a limited-resource setting.

## Figures and Tables

**Table 1:** Fluorescence intensity of 3-IS in artificial urine and artificial urine with ammonium (NH_4_^+^).

	Artificial Urine		Artificial Urine + NH_4_		% Bias
3-IS (mg/L)	Ratio	Mean	Ratio	Mean	
**1**	0.336	0.337	0.343	0.341	1.25%
**1**	0.336		0.339		
**1**	0.338		0.340		
**10**	3.402	3.400	3.344	3.339	−1.79%
**10**	3.395		3.339		
**10**	3.403		3.334		

**Table 2:** Linear regression of the relation between of 3-IS and metoprolol in artificial urine (1/x^2^ weight applied).

Curve Number	Slope	Intercept	r^2^	Range (mg/L)
1	0.4841	−0.0014	0.997	0.10–10.00
2	0.4645	0.0030	0.997	0.10–10.00
3	0.4212	0.0036	0.999	0.10–10.00
4	0.4115	−0.0012	0.997	0.10–10.00

Abbreviations: r^2^: Correlation coefficient.

**Table 3:** Intra- and inter-day accuracy and precision for 3-IS.

			Intra-Day Precision and Accuracy			Inter-Day Precision and Accuracy		
Level	Nominal 3-IS Conc. (mg/L)	Day	Mean Observed Conc. (mg/L)	Precision (%)	Accuracy (%)	Mean Observed Conc. (mg/L)	Precision (%)	Accuracy (%)
L-LOQ	0.10	1	0.105	0.47	105.3			108.4
		2	0.113	1.37	112.6	0.108	3.10	
		3	0.107	1.47	107.3			
M-QC	1.00	1	1.037	1.33	103.7			
		2	1.026	1.23	102.6	1.021	1.60	102.1
		3	1.016	0.58	101.6			
U-LOQ	10.00	1	10.105	0.20	101.1			
		2	10.008	0.33	100.1	10.047	1.70	100.5
		3	10.261	0.33	102.6			

Abbreviations: Conc.: concentration.

**Table 4:** Recovery for 3-IS spiked healthy volunteer samples.

Sample	3-IS Concentration before Spiking (mg/L)	%CV	Nominal Spiking Level (mg/L)	3-IS Urine Concentration after Spiking (mg/L)	%CV	Recovery (%)
HV 1	3.65	0.02	0.1	3.74	0.15	92.7
			0.5	4.11	0.18	96.9
			1	4.61	0.12	95.9
HV 2	3.13	0.18	0.1	3.22	0.08	92.1
			0.5	3.59	0.17	96.1
			1	4.03	0.06	90.0

Abbreviations: HV: human volunteer. Concentrations of 3-IS in urine correspond to those after 1/20 dilution of the sample as described in Methods for sample treatment. Mean 3-IS spiking concentrations (mg/L) were obtained from triplicates after interpolating in the calibration, and the obtained values were as follows: for spiking 0.1 mg/L level: 0.099 mg/L (%CV: 0.40); 0.5 mg/L level: 0.49 mg/L (%CV: 0.24); 1 mg/L level: 1.02 mg/L (%CV: 0.12).

**Table 5:** Short and medium and ISu stability at various conditions.

Condition	Time	Parameter			Sample		
			L-LOQ	M-QC	U-LOQ	HV 1	HV 2
**Benchtop**	0 h	3-IS (mg/L)	0.101	0.995	10.205	76.268	69.485
		Precision %	0.35	0.04	0.29	5.15	1.48
	1 h	3-IS (mg/L)	0.098	1.046	9.994	71.865	68.387
		Precision %	0.37	0.25	1.49	1.48	0.55
		% vs. t0	97.0	105.1	97.9	94.2	98.4
	2 h	3-IS (mg/L)	0.086	0.989	9.485	70.671	67.600
		Precision %	0.62	0.21	0.29	0.16	0.14
		% vs. t0	85.1	99.4	92.9	92.7	97.3
	4 h	3-IS (mg/L)	0.100	0.968	9.434	69.984	68.912
		Precision %	0.07	0.06	0.14	0.06	0.23
		% vs. t0	99.0	97.3	92.4	91.8	99.2
	6 h	3-IS (mg/L)	0.102	0.986	9.975	70.903	67.794
		Precision %	0.25	1.86	0.23	0.21	0.43
		% vs. t0	100.1	99.1	97.7	93.0	97.6
	7 days	3-IS (mg/L)	0.093	0.946	8.953	81.784	70.484
		CV%	0.24	0.02	0.21	0.17	0.07
		% vs. t0	92.1	95.1	87.7	107.2	101.4
	14 days	3-IS (mg/L)	0.093	0.891	8.636	87.702	77.221
		Precision %	0.24	0.68	0.05	0.27	0.6
		% vs. t0	92.1	89.5	84.6	115.0	111.1
4 °C	0 h	3-IS (mg/L)	0.101	0.995	10.205	76.268	69.486
		Precision %	0.35	0.04	0.29	5.15	1.48
	1 h	3-IS (mg/L)	0.112	1.086	9.892	79.635	71.544
		Precision %	0.29	0.23	0.45	0.38	0.24
		% vs. t0	110.9	109.1	96.9	104.4	102.03
	2 h	3-IS (mg/L)	0.108	1.008	9.262	81.194	69.345
		Precision %	0.03	0.15	0.25	0.10	0.02
		% vs. t0	106.9	101.3	90.7	106.5	99.8
	4 h	3-IS (mg/L)	0.110	0.995	9.473	80.498	70.346
		Precision %	0.04	0.08	0.98	0.39	0.47
		% vs. t0	108.9	100.0	92.8	105.5	101.2
	6 h	3-IS (mg/L)	0.112	1.039	10.099	83.783	73.008
		Precision %	0.47	0.31	0.42	1.06	0.39
		% vs. t0	110.9	104.4	99.0	109.9	105.1
	7 days	3-IS (mg/L)	0.100	1.014	9.227	81.169	71.142
		Precision %	0.59	1.23	0.27	0.65	0.46
		% vs. t0	99.0	101.9	90.4	106.4	102.4
	14 days	3-IS (mg/L)	0.108	0.940	9.347	80.680	70.085
		Precision %	0.75	0.45	0.77	0.49	0.63
		% vs. t0	106.9	94.5	91.6	105.8	100.9

Abbreviations: HV, human volunteer; ISu: 3- indoxyl sulfate.

**Table 6:** Long storage stability.

Condition	Time	Parameter			Sample		
			L-LOQ	M-QC	U-LOQ	HV 1	HV 2
**−20 °C**	**1 month**	ISu (mg/L)	0.091	0.973	10.14	85.83	70.531
		Precision%	1.06	0.41	0.12	0.12	0.06
		% vs. t0	90.1	97.8	99.4	112.5	101.5
	**2 months**	ISu (mg/L)	0.101	0.962	9.939	79.157	68.649
		CV%	0.9	0.7	0.26	0.12	0.2
		% vs. t0	100.0	96.7	97.4	103.8	98.8

Abbreviations: HV, human volunteer; ISu: 3-indoxyl sulfate.

**Table 7:** Demographic, clinical, and pharmacological characteristics.

Patient ID	Age (Years)	Sex	Time * (Days)	Concomitant Drugs	Underlying Disease	[3-IS] (mg/L) [3-IS] (μmol_3-IS_/mmol_creat_)	
**1**	3	M	−7	ACY, CAP, CID, ITRA, PRO, TRI/SULFA, VALGA	Chronic granulomatous disease	13.6	67.9
**2**	8	F	+25	ACY, AMPT, BU, LOR, MPN, OMP, OND, TRI/SULFA, UDC, VANC	Myelodysplasia	2.0	8.3
**3**	6	M	−14	DIP, FAM, LVM, TRM/SMX	Acute myeloid leukemia	31.9	22.5
**4**	10	F	−4	FAM, HYD, TAC, VALGA, VCZ	Medullar aplasia	8.1	3.4
**5**	13	F	−8	FAM, FLU, LVM, OND, PIP/TAZO, TRI/SULFA	Acute myeloid leukemia	2.8	7.0

Abbreviations: ACY: Acyclovir; AMPT: Amphotericin, BU: Busulfan; CAP: Capsofungin; CID: Cidofovir; DIP: Diphenydramine; FAM: Famotidin; FLU: Fluconazole; HYD: Hydrocortisone; ITRA: Itraconazole; LVM: Levomeprazine; LOR: Lorazepam; MPN: Meropenem; OMP: Omeprazole; OND: Ondansetron; PIP/TAZO: Piperacillin/tazobactam; PRO: Probenecid; TRM/SMX: Trimethropim/Sulfamethoxazole; TAC: Tacrolimus; UDC: Ursodesoxycholic acid; VALGA: Valgancyclovir; VANC: Vancomycin; VCZ: Voriconazole. * Time with respect to HSCT.

## Data Availability

All data are published in the article.
